# Conifer-Derived Metallic Nanoparticles: Green Synthesis and Biological Applications

**DOI:** 10.3390/ijms21239028

**Published:** 2020-11-27

**Authors:** Kanchan Bhardwaj, Daljeet Singh Dhanjal, Anirudh Sharma, Eugenie Nepovimova, Anu Kalia, Shabnam Thakur, Sonali Bhardwaj, Chirag Chopra, Reena Singh, Rachna Verma, Dinesh Kumar, Prerna Bhardwaj, Kamil Kuča

**Affiliations:** 1 Department of Botany, School of Biological and Environmental Sciences, Shoolini University of Biotechnology and Management Sciences, Solan 173229, Himachal Pradesh, India; kanchankannu1992@gmail.com (K.B.); shabnamthakur780@gmail.com (S.T.); rachnaverma@shooliniuniversity.com (R.V.); 2 Department of Biotechnology, School of Bioengineering and Biosciences, Lovely Professional University, Phagwara 144411, Punjab, India; daljeetdhanjal92@gmail.com (D.S.D.); sonali.bhardwaj1414@gmail.com (S.B.); chirag.18298@lpu.co.in (C.C.); reena.19408@lpu.co.in (R.S.); 3 Department of Chemistry, School of Chemistry, Shoolini University of Biotechnology and Management Sciences, Solan 173229, Himachal Pradesh, India; anirai3024@gmail.com; 4 Department of Chemistry, Faculty of Science, University of Hradec Kralove, 50003 Hradec Kralove, Czech Republic; eugenie.nepovimova@uhk.cz; 5 Electron Microscopy and Nanoscience Laboratory, Punjab Agricultural University, Ludhiana 141004, Punjab, India; kaliaanu@pau.edu; 6 School of Bioengineering and Food Technology, Shoolini University of Biotechnology and Management Sciences, Solan 173229, Himachal Pradesh, India; dineshkumar@shooliniuniversity.com; 7 Biomedical Research Center, University Hospital Hradec Kralove, 50005 Hradec Kralove, Czech Republic

**Keywords:** anticancer, antimicrobial, antioxidant, catalytic, conifer extract, green synthesis, metallic nanoparticles, thrombolytic

## Abstract

The use of metallic nanoparticles in engineering and biomedicine disciplines has gained considerable attention. Scientists are exploring new synthesis protocols of these substances considering their small size and lucrative antimicrobial potential. Among the most economical techniques of synthesis of metallic nanoparticles via chemical routes, which includes the use of chemicals as metal reducing agents, is considered to generate nanoparticles possessing toxicity and biological risk. This limitation of chemically synthesized nanoparticles has engendered the exploration for the ecofriendly synthesis process. Biological or green synthesis approaches have emerged as an effective solution to address the limitations of conventionally synthesized nanoparticles. Nanoparticles synthesized via biological entities obtained from plant extracts exhibit superior effect in comparison to chemical methods. Recently, conifer extracts have been found to be effective in synthesizing metallic nanoparticles through a highly regulated process. The current review highlights the importance of conifers and its extracts in synthesis of metallic nanoparticles. It also discusses the different applications of the conifer extract mediated metallic nanoparticles.

## 1. Introduction

Nanobiotechnology has had an enormous impact on all life forms, which has intrigued researchers currently [1]. Richard Feynman a physicist, in the year 1959 described the theoretical concept of miniaturization with hidden hints for nanotechnology for the first time, which implies a technological advancement utilizing materials with dimensions of about 1–100 nm [2,3]. Nanoparticles (NPs) have diverse applications in agriculture, biomedicine as antimicrobial agents, catalysis, biolabeling, sensors, electronics, fiber optics and other areas [4]. NPs bear properties that are different as compared to their bulk materials due to variation in their shapes, particle size and increased surface area. Studies on biomedical science invigorate interest in identifying the application of NPs in varied fields. The NPs can be synthesized, and stabilizing formulation can be developed through chemical and physical synthesis methods [5]. However, the use of environment-corrosive chemicals in the former synthesis process has ecodeterring potentials and can lead to the generation of toxic byproducts. Therefore, the desirability of the green synthesis approaches for NPs involves superior control over no or decreased use of ecotoxic chemicals [6]. Nanoparticles of desired size shape and functionality can be generated through two different fundamental approaches, viz., bottom-up and top-down methods [7]. Previously, nanomaterials/nanoparticles were synthesized using physical forces/agencies, such as ball milling, etching, lithography and sputtering [8].

The continuous developments in the timber industry have compelled scientists to opt for biological approaches to protect the environment. Generally, the natural ecofriendly synthesis of NPs involves the utilization of plant extracts and microbes [9,10,11,12]. These NPs can be prepared under different synthesis conditions and characterized by various analytical techniques [13]. The NPs derived from plant extracts dominate over NPs synthesized from microorganisms as the former is synthesized via a single-step, non-hazardous method. This type of synthesis is cost-effective and eco-friendly [7]. The conifer trees produce a broad spectrum of secondary metabolites, including polyphenols and terpenes, to overcome stress conditions [14]. These secondary metabolites have the potential to reduce metal ions into NPs [7]. This review intends to summarize the literature related to metallic NPs synthesized via conifers extracts, characteristics of the synthesized NPs and their biological properties like anticancer, antioxidant, antimicrobial, etc.

## 2. Importance of Conifers

Conifers are evergreen woody gymnosperms characterized by single veined leaves in the form of blade, scales and needle, and unisexual female and male cones having bract scales [15]. The conifers encompass two formerly known classes or orders viz Coniferopsida (Coniferales) and Taxopsida (Taxales) but exclude other gymnosperms like *Cycads*, *Ephedras*, *Gnetums* and two unique gymnosperms *Welwitschia* and *Ginkgo.* There are eight families: Araucariaceae, Cupressaceae, Cephalotaxaceae, Pinaceae, Phyllocladaceae, Podocarpaceae, Sciadopityaceae and Taxaceae [16]. Conifers have been stated as the reservoir of different natural products like essential oils (EOs), gums, resins and terpenes [17].

Conifer trees are abundantly found all over the world and have highly diverse natural compounds. Secondary metabolites such as phenols, flavonoids, alkaloids and terpenes found different part such as in leaves, bark, stem, seed and root tissues show structural and chemical diversity [14]. Therefore, conifers are considered as an essential source of biologically active agents possessing important medicinal attributes that have been utilized for the treatment of various ailments since ancient times [18,19]. These natural substances are known to exhibit the potential to influence the growth of insects and plants [20]. Natural compounds as EOs and extracts of genera *Pinus*, *Cedrus*, *Juniperus*, *Taxus*, *Picea* and *Abies* are effective against diseases such as cancer, diabetes, asthma, liver and kidney disorders, cardiovascular-related problems and many other bacterial and fungal infections [18]. The species, *Araucaria angustifolia* and *A. excelsa*, have ethno pharmaceutical values due to the presence of bioflavonoids, lignans while *A. araucana* is rich in terpenes [21]. It has been reported that in vitro and in vivo studies of an extract compound from *A. angustifolia*, *A. bidwillii*, *Cupressus sempervirens*, *Podocarpus* spp. and *Thuja occidentalis* possess biological activities like allelopathic, antibacterial, antidiabetic, antidepressant, antiedematogenic, anti-inflammatory, antioxidant, antiproliferative, antiviral, cytotoxic and entomotoxic, neuroprotective activities, gastro protective activity, hypocholesterolemic and antifeedant activities [20,21,22,23,24,25]. Acetone, dichloromethane, methanol and ethyl acetate extracts of leaves and cones of *C. sempervirens* var. *horizantalis* (CSH) and var. *pyramidalis* (CSP) exhibit inhibitory activity against tyrosinase (TYRO), butyrylcholinesterase (BChE) and acetylcholinesterase (AChE) enzymes [25].

*Thuja* (Esberitox) has been used for treating the common cold and many bacterial infections. In various in vivo and in vitro test models, *Thuja* immunopharmacological potential has also been studied [26]. In Korea, *Torreya nucifera* is known for its ethnopharmacological use against constipation, diabetes, hemorrhoids and infection caused by helminths. Additionally, in Asia, *T. nucifera* reported as having antioxidant activity (L.) seeds are used in treating various diseases [27,28]. Amentoflavone, a biflavonoid compound naturally occurring in Podocarpaceae and Cupressaceae shows good anti-inflammation, antidiabetes, antioxidation and antisenescence effects on many essential processes of the central nervous and cardiovascular system [29]. Reports suggest that *Taxus yunnanensis* extracts and their purified compounds displayed cytotoxicity against HeLa, MCF-7, HT1080 and K562 cell lines. Many researchers have found that the extracts of *T. yunnanensis* can be used as an antiosteoporotic, anti-inflammatory and hypoglycemic agent for diabetes [30]. As per the literature, it has been found that conifers are abundantly found and have much importance in our daily life [14]. So, now a day’s keeping these things in mind, new research of interest is growing to develop more uses of conifers by deriving metallic NPs, which helps in dealing problems, diseases related to human beings plants pathogens agriculture, promoting plant growth by enhancing the quality of soil and industrial uses [31,32,33,34].

## 3. Green Synthesis of Nanoparticles Mediated by Conifers Extract Synthesis Mechanism, Characterization

**Figure 1:**
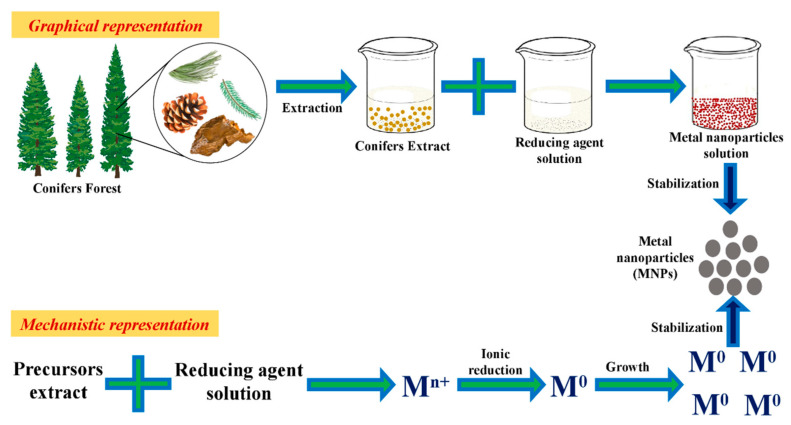
Graphical overview of the green synthesis protocol using conifer extracts for synthesizing metallic nanoparticles.

Plants are considered as bioreactors for the synthesis of metallic NPs. Different physical and chemical approaches are used to synthesize metal NPs, which allow one to obtain particles with the desired characteristics [35]. Conifers exhibit unique metal NPs synthesis properties (Figure 1). Green-synthesis of metallic NPs is an efficient, trouble-free, economical and environmental-friendly biological synthesis approach [36]. Metallic NPs include both types of pure metals and metal oxides, which have various applications [37]. The critical factor, which needs to be considered during the synthesis of NPs, is the selection of a solvent medium, which needs to be a non-toxic material for NPs stabilization and an ecofriendly reducing agent [38].

### 3.1. Mechanism

For the synthesis of metallic NPs from conifers, plant samples were collected, washed and dried to form a powder. The powdered sample was mixed with 250 mL double distilled water and boiled for 5 min. The heated materials were filtered and centrifuged to obtain a clear extract. The metallic salt solution was added dropwise into extract under sonication and then incubated for different time intervals (10–120 min), pH ranges (7–11) and temperatures. The color change indicates the formation of NPs due to the reduction of metal ions into metal atoms by excitation of surface plasmon vibrations [30].

Additionally, compounds such as polyphenolics, peptides, vitamins, sugars and water present in extracts of conifers are effective for synthesizing NPs [39,40,41,42,43,44]. Interestingly, plant metal bioaccumulation studies have shown that metals are usually aggregated in the form of NPs. The polyphenol compounds are responsible for the reduction process by the abstraction of hydrogen [35]. Further, these compounds also act as capping agents to avert the coalescence of colloidal particles through the interplay of electrostatic forces [45]. The metal ion reduction was monitored by measuring the absorbance from 300 to 600 nm using UV–vis spectrophotometer to find the absorbance peak. The solution was then centrifuged, and the pellet was dried by lyophilization [30].

### 3.2. Characterization Techniques

Metallic NPs synthesized from various conifers extracts had diverse shape, size and surface area; and were categorized using different approaches as shown in Table 1. The synthesized metallic NPs structural composition, size, shape and crystal phase were deduced using energy-dispersive X-ray spectroscopy (EDX), FTIR, SEM, HRTEM, UV–Vis [46], XRD, atomic force microscopy (AFM) and dynamic light scattering (DLS) [47,48]. The range of absorption of the UV spectra from wavelength 300 to 800 nm illustrates the existence of several metallic NPs of varying size, i.e., from 6 to 200 nm [30,47]. DLS analysis entails estimation of the size of synthesized NPs and the quantification of the charges on the surface of the NPs. The composition of the element is determined through the EDX analysis [49]. XRD is functionalized to recognize crystallite size. FT-IR spectroscopy detects the surface residues and the functional groups such as flavonoids, hydroxyls and phenols, which bond with the surface of the NPs throughout the process of the synthesis for an effective reduction and stabilization [50].

## 4. Types of Nanoparticles

### 4.1. Metal Nanoparticles

#### 4.1.1. Silver Nanoparticles

In nanotechnology, silver nanoparticles (AgNPs) are well-known. They have gained significant attention because of their extraordinary antimicrobial ability against several microorganisms and role in combating the drug resistance menace in multidrug resistant microbes. These properties of AgNPs can be effectively utilized in food packaging, agriculture for eradication/ management of crop diseases, biomedicine, drug resistivity and pharmacology to formulate novel drug carrier systems and in decontamination of water [76,77]. AgNPs derived MNPs from various conifers, and their applications are in Table 2.

Synthesis of gold and silver NPs from different plant organ extracts such as bark, needle, cone, callus and berry of a variety of coniferous plant species are shown in Table 1. Due to better adaptability and easy down streaming to nanosystems, AgNPs synthesis supports better control on the shape and dimensions of NPs [78]. In the presence of aqueous medium and oxygen, silver atoms found in Ag2O NPs release Ag+ ions. Ag+ ions are a potent oxidant, which can oxidize microbial biomolecules non-specifically. In water-treatment systems, this potential makes AgNPs, suitable alternative disinfectants [33]. However, Das et al., in 2018 reported in their studies that AgNPs are toxic to numerous organisms, mammalian cells and humans [79]. The novel AgNPs synthesis via two-step procedure initiates via the reduction of Ag+ ions to Ag, followed by aggregation and stabilization of nanoparticles [80]. The agricultural and biomedical applications of AgNPs obtained from different coniferous plants are shown in Table 2.

#### 4.1.2. Gold

Gold nanoparticles (AuNPs) display many unique biological, favorable chemical and optical properties, which make them safe for more comprehensive applications [82]. Due to their significant biocompatibility, Au NPs are widely reported in different varieties of conifers, as shown in Table 1. Many studies proved that catalytic, optical, physical and thermal properties of AuNPs depend on their shape and size, which has drawn attention towards the development of an optimized protocol for the synthesis of monodisperse AuNPs formulation. Currently, biological synthesis approach involving plant extracts without the use of toxic chemicals during synthesis has gained enormous attention due to the benefit of avoidance or minimization of adverse impact on the application [83]. Synthesis of gold NPs from different types of extracts of various conifers such as *Pinus*, *Taxus*, *Juniperus*, *Abies* and *Thuja* has been reported [54,73]. AuNPs synthesized from plant extracts exhibit several advantages with primary includes the biocompatible or less-toxic nature of the generated NPs. Moreover, the therapeutic potential of drugs can be potentially enhanced by significantly reducing the particle size to the nanoscale. Use of such formulations will enhance the interaction of the active drug molecule with a specific protein and increases the efficiency of the formulated drug [84]. High purity AuNPs can be utilized for anticancer applications and also have the potential to bind to the human serum albumin (HSA) protein [85]. Application of gold NPs synthesized from various conifers is shown in Table 2.

### 4.2. Nanoparticles of Metallic Oxides

Nanoparticles of zinc oxide (ZnO NPs) are non-toxic, biosafe, ecofriendly and have an easy amendable nature that makes them the targeted candidate for biological applications [86]. Zn NPs derived from plant extract have been applied for varied biomedical benefits such as the treatment of chronic disorders like cancers, and diabetes, as a potent antimicrobial agent to kill pathogenic bacteria, fungi and for use as a photocatalyst for decontamination of contaminant dyes from water and sediments [72,87,88]. Additionally, studies suggested that Zn NPs have significance in eradicating hardy, persistent aquatic weed resistant to chemical and physical means of eradication. *Taxus baccata* extracts have been used to synthesize Zn NPs. UV–Vis spectrophotometry analysis of the *T. baccata* extracts derived Zn NPs exhibited in the range of 250–400 nm. Zn NPs obtained were crystalline and hexagonal shaped, showing an average particle size of 20–25 nm [72].

Copper nanoparticles (CuNPs) have unique properties like high chemical reactivity, mechanical strength and antimicrobial potential, which made them useful for diverse applications. Additionally, due to a high surface area–volume ratio, CuNPs can easily interact and react with other NPs [89]. Studies revealed excellent antibacterial activity of Cu-NPs derived from *P. merkusii* flower extract against *B. subtilis* and *E. coli* as compared to Ag-NPs [53].

**Figure 2:**
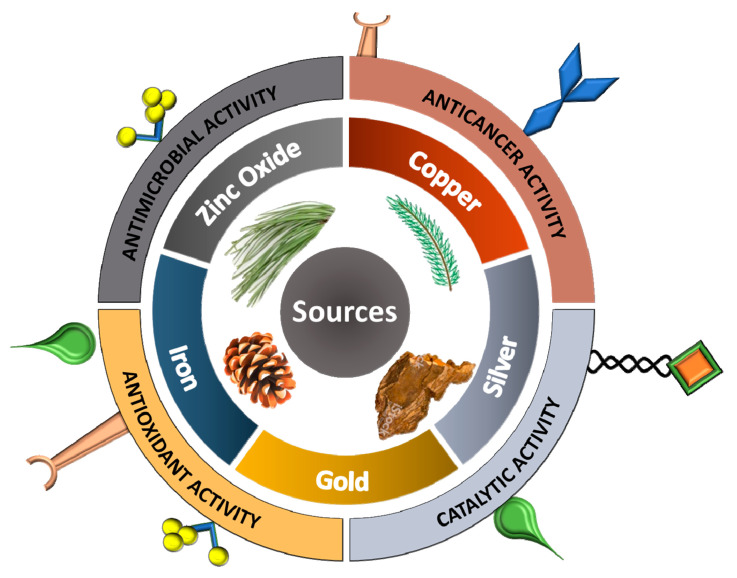
Graphical illustration of conifer sources used for the production of metallic nanoparticles with potential biological activities.

Iron nanoparticles (FeNPs) have unique biological and physicochemical characteristics, which make it the most applicable nanostructures in a different field of science. Additionally, FeNPs are biocompatible and easy to handle and have sturdy magnetic properties. FeNPs synthesized from *P. eldarica* needle extract had a size range of 8–34 nm [61]. FeNPs has transpired antibacterial characteristics against bacterial strains like *B. subtilis*, *S. aureus*, *E. coli*, *P. aeruginosa*, *K. pneumonia* and *Streptococcus pyogenes* [33]. In environmental sciences, FeNPs have been used both as a catalyst and nanosorbent [90]. Additionally, they are efficient in removing and detoxifying chemical toxic agents and organic pollutants like arsenic, chromium, carbon monoxide, mercury and toxic ions [91,92,93,94]. Additionally, FeNPs have been stated to be operative as a Fenton-like catalyst for the removal of contaminant toxic dyes from aqueous environments [95]. Metal derived NPs and their application in are presented in Figure 2.

## 5. Conifer-Derived Metallic Nanoparticles Potential Applications

### 5.1. Biomedical Applications

#### 5.1.1. Antimicrobial Action

Metal NPs synthesized from different conifer extracts possess good antibacterial, antifungal and insecticidal potentials. Plant extracts show mild activity against some Gram-positive and Gram-negative bacteria and fungi. At the same time, MNPs synthesized from plant extracts are known to exhibit high potential against yeast and Gram-positive and Gram-negative bacteria. It also has been reported that MNPs derived from conifers found in high altitude show good fungal and bacterial inhibitory activity [65].

##### Mechanism of Action Towards Microbes

**Figure 3:**
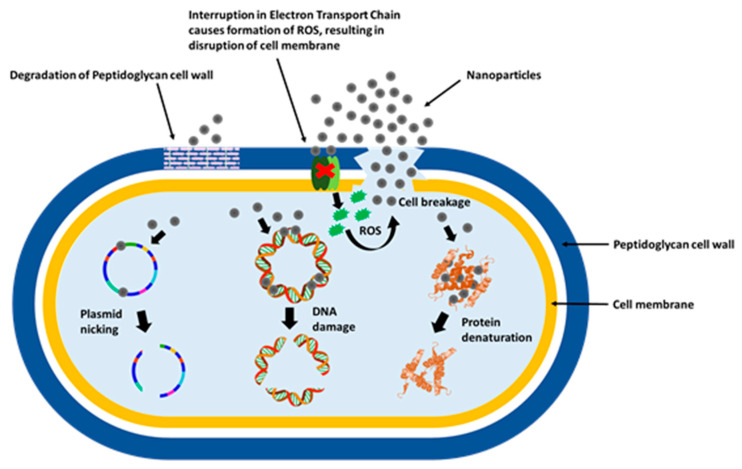
Graphical illustration of the antimicrobial mechanism of nanoparticles.

Despite several approaches, technologies and formulations had been made over the years; still, the precise mechanism of action of MNPs toward bacteria and fungi has not been fully understood. However, several reports are available in the literature, which comes in favor that the mechanism of action of MNPs in bacteria and fungi is almost similar. The antimicrobial action of MNPs is related to the following four-step mechanism; (a) direct contact with the cell membrane, (b) destabilization of the cell membrane, change in its permeability and release of metal ions, (c) production of reactive oxygen species (ROS) and free radicals and (d) signal transduction pathways modulation (Figure 3) [96].

##### Direct Contact with the Cell Membrane

The size and zeta potential of the MNPs influence their adhesion on the bacterial surface. Depending on the technique used for their fabrication, MNPs may possess positive, negative or neutral charges. Since both bacteria and fungi have a partial negative charge on their surfaces, positively charged MNPs gets strongly attracted to the negatively charged cell surface, resulting in improved antimicrobial activity as compared to negatively charged or neutral MNPs [97].

##### Destabilization of the Cell Membrane, Changes in Its Permeability and Metal Ions Release

After adhesion, MNPs with smaller size penetrate directly into the cell, whereas larger NPs are retained outside the cell surface. In both cases, MNPs continue to release their ions and destabilize cell membrane. Cell wall destabilization causes bacterial permeability, allowing entry of large-sized NPs into the cell. Inside the cell, improved production of ions leads to interaction with proteins, lipids, and DNA, resulting in cell dysfunction and cell death [98].

##### Production of Reactive Oxygen Species (ROS) and Free Radicals

Furthermore, MNPs are well known to produce ROS and free radicals such as H_2_O_2_, O_2_^−^ and OH^•^. The ROS and free radicals are highly reactive species that are responsible for the production of extreme oxidative stress inside the cell resulting in cell apoptosis, proteins destruction, DNA damage and finally cell death [99].

##### Signal Transduction Pathways Modulation

It has been noticed that under normal conditions, ROS occurs naturally in microbes. Antioxidant enzymes such as glutathione (GSH), superoxide dismutase and catalase protect the microbes from the effects of ROS. These enzymes are efficient in eliminating some toxic species at a low concentration, but when the oxidative stress becomes high, they are rendered ineffective in neutralizing toxic species at a high concentration. At high concentration, oxidative species interact with respiratory proteins and inactivate these antioxidant enzymes because of their high affinity toward thiol, carboxyl and phosphates groups present in respiratory proteins [100].

Reported study on antimicrobial (bacteria and fungi) activity by different parts of the conifers derived MNPs were discussed. The antibacterial activity of *Picea abies* bark extract synthesized AgNPs was investigated in vitro against *E. coli*, *K. pneumoniae* and *P. aeruginosa* with an minimum inhibitory concentration (MIC) of 3.25, 3.25 and 7.5 mg/mL [101]. AgNPs synthesized from *T. yunnanensis* callus had a significant growth inhibitory activity against human pathogenic bacteria with an MIC of 8 for *E. coli*, 1 for *B. subtilis*, 1 for *S. aureus* and 4 μg/mL for *S. paratyphi*, however, the growth of bacteria was inhibited by AgNPs 2 µg/mL [30]. The MIC values shown by AgNPs obtained from the cone extract of *Pinus thunbergii* were low for the Gram-negative bacterial strains, *Xanthomonas oryzae*, *Burkholderia glumae* and *Pseudomonas syringae* were 11.9 and 6 μL/mL respectively while the MIC for the Gram-positive *Bacillus megaterium* and *B. thuringiensis* were 11.9 μL/mL [81]. The *Pinus densiflora* cone extract mediated AgNPs at a working concentration of 40 µg per mL effectively inhibited skin pathogen bacteria such as *Brevibacterium linens* (zone of inhibition: 7 mm) compared to commercial AgNPs *Propionibacterium acnes* (14 mm), *Staphylococcus epidermidis* (10 mm) and *B. cereus* (9 mm) [56]. The antibacterial activity was evaluated on *S. aureus* by aqueous extract, and copper NPs derived from the *P. merkusii* cone. The bacterial growth–inhibition was recorded to be substantially high (29.67 ± 0.01 mm) at 100% concentration [53]. The researcher advocated the use of this formulation as a natural fungicide. AgNPs mediated from the gum of *A. heterophylla* have been reported to be effective against Gram-positive bacteria *Streptococcus* sp. [48].

The zone of inhibition of AgNPs by leaves extract of *C. torulosa* against *B. subtilis* was observed and found to be 5 mm for 50 µL, 6 mm for 100 µL, 6 mm for 150 µL and 3 mm for 200 µL whereas *Salmonella enterica* showed 6 mm for 50 µL, 7 mm for 100 µL, 8 mm for 150 µL and 10 mm for 200 µL. AgNP’s showed the zone of inhibition against *P. aeruginosa* as 4 mm for 50 µL, 6 mm for 100 µL, 6 mm for 150 µL and 6 mm for 200 µL [64]. Few researchers have stated that *Juniperus procera*; obtained from a high altitude possess both antibacterial and antifungal activity [102]. *J. procera* leaf extract derived AgNPs showed antibacterial and antifungal activity against *B. subtilis*, K. *pneumonia*, *Micrococcus luteus*, *Proteus mirabilis* and *C. albicans* with the formation of inhibition zones of 28 ± 1.2, 18 ± 0.9, 28 ± 1.1, 29 ± 1.3 and 24 ± 0.1.2 mm respectively [65]. Studies reported that *T. occidentalis* mediated AgNPs exhibited an inhibitory effect on *B. subtilis*, *Listeria monocytogenes*, *P. aeruginosa*, *Salmonella typhimurium* and *S. aureus* at 5–10 g/mL concentrations. The AgNPs biosynthesized by using *T. occidentalis* (L.) leaves extract exhibits improved inhibitory activity against microbes [70]. The AgNPs concentration 30 µg/mL made an inhibitory growth zone for *B. subtilis* (15.6 mm), *E. coli* (16 mm) and *Pseudomonas putida* (16.3 mm). Besides, at a concentration of 10 µg/mL growth of all the fungal strains used in the study was considerably reduced with the medium showing no signs of proliferation. The study showed that these biosynthesized AgNPs could serve as antibiotic agents and can be used in combination with other antibiotics [66]. Studies suggested that the leaves extract of *T. occidentalis* synthesized AgNPs showed significant larvicidal activity against the *Culex quinquefasciatus* mosquito with an LC_50_ value of 39.90 ppm and LC_90_ value of 129.48 ppm. Additionally, as it is dose-dependent, the increase in concentration from 100 to 300 ppm increased the mortality rate to 100% after 72 h of treatment [68]. Pine pollen derived AgNPs efficiently exhibited quantitative inhibition and disruption of growth of fungus *Neofusicoccum parvum* [103]. AuNPs synthesized from *P. kesiya* pollen exhibited fungicidal activity against *Candida albicans*, at 500 μg/mL concentration with 17.51 zones of inhibition [73]. The AgNPs generated from the *P. wallichiana* stem displayed good antibacterial activity than antifungal activity against *Acinetobacter baumannii*, *E. coli*, *Morganella morganii*, *Proteus vulgaris*, *P. aeruginosa* and *S. aureus*. *A. baumannii* showed activity 60% with minimal inhibitory concentration (MIC) of 2.36 mg/mL and minimal bactericidal concentration (MBC) of 5.0 mg/mL. Additionally, AgNPs showed moderate antifungal potential against *Aspergillus niger* (40%), *Penicillium notatum* (45%) and *P. chrysogenum* (50%), whereas it displayed minimum activity against *A. parasiticus* (13%), *Hemimycena pseudocrispula* (30%) and *Verticillium longisporum* (21%) [74].

#### 5.1.2. Anticancer

According to the WHO (World Health Organization) report of 2018, cancer has emerged as a leading cause of death, accounting for an estimated death of 9.6 million people globally. In males, cancers such as colorectal, liver, lung, prostate and stomach are predominant while cervical, breast, colorectal, lung, and thyroid cancer are most common in females [104]. Different types of natural bioactive compounds present in plants are believed to have medicinal value and are potential candidates to develop anticancer drugs. Various classes of heterocyclic compounds (alkaloids and flavonoids) have been isolated from several conifer plants showing cytotoxic efficacy against multiple types of cancerous cells both in vitro and in vivo [74,105]. The field of nanotechnology holds the potential to transform cancer diagnostic methods and therapeutic technologies [106].

##### Mechanism behind anticancer activity

**Figure 4:**
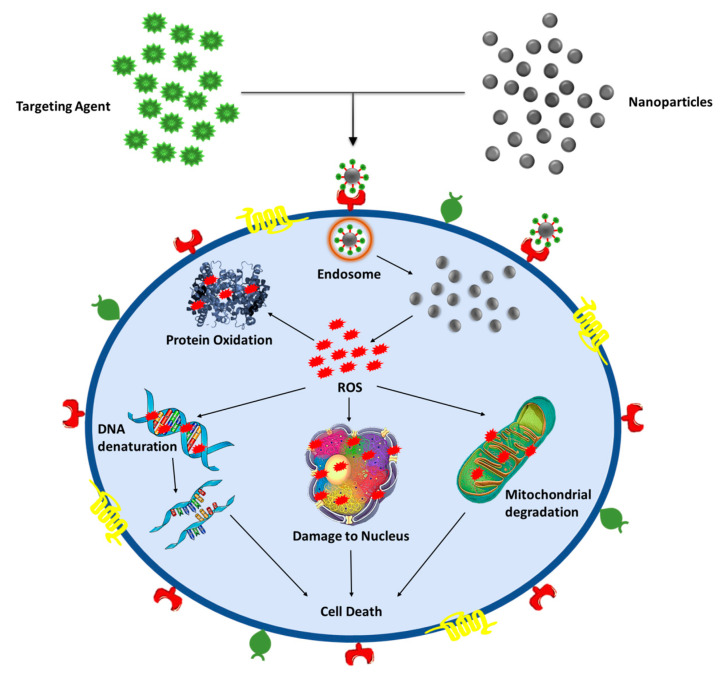
Graphical illustration of the anticancer mechanism of nanoparticles.

In literature, numerous reports are available describing the mechanism behind the action of MNPs toward cancer cells (Figure 4). However, the precise mechanism of anticancer activity is till not well understood. It is suggested that the interaction between MNPs and cancer cells may take place in various ways like electrostatic interaction between the cell surface and metallic nanoparticles, nanoparticles captured by cell receptors and internalization of nanoparticles via endocytosis. It is well known that MNPs can easily generate reactive species inside the cell system leading to DNA damage, mitochondrial disruption, protein oxidation and, finally, cell death [106,107]. Additionally, the ability of MNPs to generate the hyperthermia effect inside the cancer cell functions as a drug carrier and regulate pH-dependent release makes it a superior candidate for anticancer activity [108].

Cancer is a lethal threat to humankind [109,110]. Regrettably, despite the success in designing various anticancer therapeutics, this disease remains relentless because of the toxic nature of the drugs towards normal cells. Therefore, the treatment of cancer with the help of NPs can be an effective approach. The nanosize of NPs makes them distinctive and reconcilable for penetration within the cells. This attribute of NPs contributes to its efficacy against tumor cells even at a low dosage while also reducing the toxic effects to the adjacent normal cells [101]. NPs derived using conifer extracts have gained significant attention due to their adaptable properties and easy synthesizing procedure [78].

The pine barks extract derived AuNPs have shown concentration-dependent cytotoxicity towards non-malignant HEK293 kidney cells and malignant A549 lung cells with a decrease in cell viability to 23% and 35% respectively. Additionally, it also increases the death rate of lung cancer cells and reduces the toxic effects on non-malignant human embryonic kidney cells. Whereas, pine bark derived oleamide capped AuNPs have been reported to decrease the viability of the cancerous cells with an insignificant cytotoxic effect on non-malignant cells. Thus, the pine extracts mediated AuNPs exhibited the potential of anticancer agents [85]. Studies have unveiled that *Pinus sylvestris* bark extract contains high amounts of phenols and shows cytotoxic effects towards HeLa cells by inducing apoptosis after 48 h of exposure [111]. Additionally, the biosynthesized AgNPs from callus of *T*. *yunnanensis* showed significant inhibition in a dose-dependent manner in four human malignant cells. For the A549 cell line, the half maximal inhibitory concentration i.e., IC_50_ of AgNPs was recorded to be 40.3 µg/mL while it was reported to be 42.2 µg/mL for the MCF-7cell line, which indicated a moderate cytotoxic effect on both the cell lines [30]. On the increase in concentration from 0.2 to 1 mg/mL of biologically synthesized AgNPs using the gum of *A. heterophylla* has been reported to reduce the cell viability rate of the MCF 7 cell line [48]. ZnNPs synthesized from *T. baccata* showed a cytotoxicity effect on the breast cancer cell line. Zn has the efficiency to prevent cell change to cancer cells. The MTT test by the MCF-7 cell line proved that the anticancer activity of Zn nanoparticles from *T. baccata* leaves ethanolic and alcoholic extract was expected to be higher than that of extract alone as the ZnNPs concentration up to 50 mM decreased cell viability (24.83%) with a maximum cell inhibition of 75.17% [72]. It has been found that AgNPs synthesized from the aqueous extract of *T. baccata* needles having an IC_50_ value of 0.25 µg mL^-1^ showed significant mortality of malignant cells of the MCF-7 cell line after 48 h of exposure. The cytotoxic effect of NPs substantially improved with the increase in dosage and incubation time with the killing of more than 50% of the cells after exposure for 72 h [34]. Additionally, it has been reported that the *T. baccata* needles ethanolic extract synthesized AuNPs displayed gradually higher anticancer activity on all the three cancer cell lines, i.e., MCF7, HeLa and Caov-4 in contrast to the aqueous extracts derived AuNPs [59].

*T. occidentalis* leaves mediated AgNPs have also been accorded for anticancer potential against HeLa, MDA-MB 231, MCF 7 and KB cell lines at a dosage of 6.25–50 g/mL. AgNPs exhibited 45% cell death in peripheral blood mononuclear cells (PBMCs), which is significantly less than the cytotoxicity exhibited in the anticancer study with an equal dose. Cytotoxicity of *Thuja* extracted AgNPs was significantly much higher in cancer cells compared to other normal cells [70]. The MTT assay showed that the antiproliferative action of the *T. occidentalis* leaves AgNPs against the HeLa cell line is dose-dependent. However, with the increase in AgNPs concentration, there was a gradual appearance of toxicity. Furthermore, Coincubation of the AgNPs with the concentration of 50 g/mL results in the reduction of 70% viability of the cancerous cells. Whereas, treating by AgNPs up to a 50 g/mL concentration on L929 cells showed no statistically significant change in cell proliferation. However, on the elevation of AgNPs concentration to 65 g/mL showed a significant reduction in cell viability [69].

#### 5.1.3. Antioxidant

**Figure 5:**
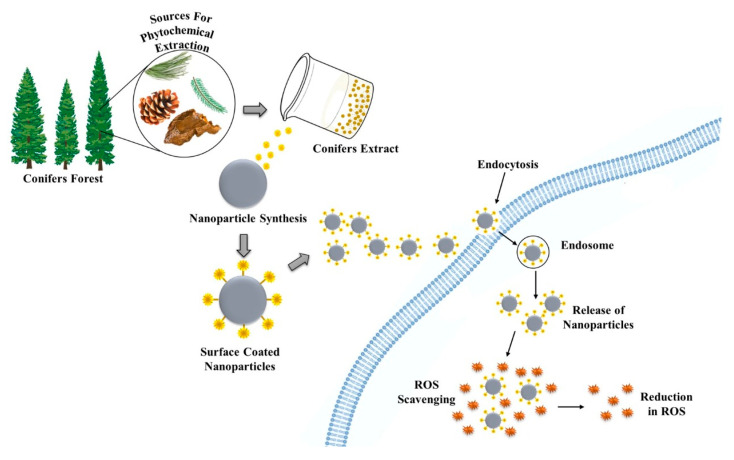
Graphical illustration of the antioxidant mechanism of nanoparticles.

Reactive oxygen species (ROS) such as superoxide dismutase, hydrogen peroxides and hydrogen radicals are highly reactive and toxic species, overproduction of which may cause damage to lipids, DNA, carbohydrate, proteins and build oxidative stress, thereby resulting in the formation of different diseases [112]. Antioxidants molecules have emerged as potential candidates that scavenge these free radicals and ROS and prevent associated problems [113]. MNPs act as an antioxidant agent via two possible mechanisms; (1) via single electron transfer and (2) hydrogen transfer. Single-electron transfer refers to the reduction of oxidative compounds by an electron donation, whereas hydrogen transfer includes total oxyradical scavenging using a hydrogen atom [114,115]. During the oxidation process, the generation of free radicals takes place in the cells. The formation of free radicals disturbs the normal cellular processes and causes cellular damage (Figure 5). Antioxidants are required to prevent cellular, and DNA damage and oxidative stress besides help prevent chronic disorders such as cancer, malignant transformations and heart diseases [116].

The AgNPs mediated from *P. abies* stem bark extract possessed free radical scavenging activity against both 2, 20-azino-bis (3-ethylbenzothiazoline-6-sulphonic acid (ABTS) and DPPH radicals [51]. The study revealed that instead of metal oxides, the noble metal NPs (AgNPs and AuNPs) are strong scavengers of superoxide ions, nitric oxide radicals, DPPH and hydroxyl radicals. The biosynthesized AgNPs from the *P. wallichiana* stem has been stated to have the proficient antioxidant potential of 70.25 ± 0.56% and 61.77 ± 0.828% at a concentration of 400 and 500 µg/mL respectively [74].

### 5.2. Other Activities

Different development parameters like biomass accumulation, growth, germination and biochemical activities have been evaluated in plants, which shows varied effects of these metallic NPs in diverse plants [37]. Zero valent iron coated silver nanoparticles (ZVI@AgNPs derived using leaf extract of *C. sempervirens* have been reported to show significant catalytic potential as they can remove 98.5% initial dye in 4 h [33]. Decolorization of Congo red, Orange G and Rhodamine B dyes have been reported by use of AgNPs derived using *Cupressus torulosa* D.Don leaf extract [64]. Green synthesized MNPs derived using *T. occidentalis* leaves (GSNPs) have demonstrated considerable improvement in plant growth and soil quality. Total nitrogen, and nitrate leaching assays, revealed that GSNPs reduces the leaching of nitrate from the soil, which substantially improves the total nitrogen (N) content. The GSNPs amended soil has shown an adequate yield of *Proteus vulgaris.* Moreover, high chlorophyll content and enzyme activation have been recorded in GSNPs exposed plants. Of GSNP a 50 mg kg^−1^ concentration has been claimed to be advantageous for improving plant growth and soil quality. However, a higher dosage of green synthesized nanoparticles (GSNPs) might be detrimental to plants. *T. occidentalis*-derived NPs have been reported to enhance the N availability in soil, thus significantly advancing the nitrogen-driven growth potential of crop plants by acting as growth promoters [79]. Pine needle extract mediated palladium nanoparticles (PdNPs) have been developed in which palladium/residue of the pine needle (Pd/RPN) are reused as a catalyst. The Pd/RPN nanocomposite shows high catalytic activity for the Suzuki coupling reaction. Moreover, the catalyst can be easily recovered through centrifugation and reutilized for at least six-folds without losing its viability [32].

Clotting of blood within the blood vessels often leads to thrombotic diseases such as myocardial infarction. Diverse agents are employed to dissolve blood clots in vessels, which may otherwise have serious and fatal consequences. Studies have reported that *P. wallichiana* stem mediated AgNPs shows effective thrombolytic activity in contrast to the crude extract. The thrombolytic potential of crude leaves extract, and AgNPs exclusively were 15.9% and 25.8%, respectively [74]. The *P. wallichiana* stem mediated AgNPs have also shown a significant antipyretic effect in mice after 1, 2 and 3 h of administration. AgNPs cause the reduction in temperature (36.40 ± 3.11°C at 10 mg/kg) and bodyweight of mice in contrast to paracetamol (50 mg/kg) [74].

## 6. Conclusions and Future Outlook

Nature has an ingenious way to synthesize highly effective miniature functionalized materials. Additionally, the increasing interest towards green chemistry and its utilization for synthesizing metallic nanoparticles lead an aspiration to develop an economical and environment-friendly approach. The primary benefit of synthesizing these metallic nanoparticles with conifer plant extract is that it is cost-effective, economical, energy-efficient and healthier, as it protects the environment and human health by producing less waste and safe products. Besides, the synthesis of metallic nanoparticles from conifer extracts has an advantage over other biological entities such as microbes, which require maintenance of culture and consume a lot of time. Moreover, microbes also lose their ability to synthesize nanoparticles with time. Hence, metallic nanoparticles synthesized via plant extract will have an immense impact in the coming future. Green synthesized metallic nanoparticles have significant features of nanotechnology with unmatched applications.

Various reports have been recorded about the synthesis of metallic nanoparticles using conifer extracts. However, there is a need to explore commercial, economic and ecofriendly approaches. Moreover, reproducibility of NPs in a high amount also poses a challenge in the green synthesis of conifer-derived MNPs. It has been observed that there is substantial variation in the composition of conifer extracts of the same species when procured from different regions, which is an attribute for the different result during in vitro evaluation. Moreover, subsequent interaction with metal ions is believed to contribute to the variability in size and shape of nanoparticles. This imposes a major challenge for the synthesis of metallic nanoparticles with the help of conifer extracts as the stabilizing and reducing agent. Therefore, the selection among plants with the season in which the biological material is collected is an essential requirement aiming the reproducibility of the synthesis. Additionally, in MNP synthesis, the reaction time and temperature influence the shape and size of synthesized MNPs. Many studies do not report the time of the year when the plant material for the MNPs synthesis is collected, the conditions of growth wherein the plant was cultivated or collected, and the quantification of major metabolites present in the extract. All that information must be considered essential for the reproducibility of the process and to understand the mechanisms culminating in the formation of MNPs. Furthermore, there is a need to identify biomolecules responsible for the synthesis of metallic nanoparticles and develop a single-step method to surpass the above-discussed challenges and pave the way for new opportunities for green chemistry to create ecofriendly metallic nanoparticles.

## Figures and Tables

**Table 1:** Green synthesis of metallic nanoparticles synthesized from conifer extracts and their characterization through different analytical techniques.

Types of Conifer	Family	Parts of Plant Used	Types of Metallic NPs	Reducing Agent	Stabilizing Agent	Reaction Time	Reaction Temp	Characterization	Shape	Size (nm)	Stability	Ref.
*Picea abies* L.	*Pinaceae*	Bark	Silver	Phenolic compounds	Phenolic compounds	3 h	60 °C	FTIR, UV–Vis, TEM	Sphere and Polygonal	44	ND	[51]
*Pinus eldarica*	*Pinaceae*	Bark	Silver	Bark extract	Bark extract	ND	RT	UV–Vis, TEM	Sphere	10–40	ND	[52]
*Taxus yunnanensis*	*Taxaceae*	Callus	Silver	Callus extract	Callus extract	120 min	NS	XRD, TEM, FTIR,	Sphere	6.4–27.2	More than 2 weeks	[30]
*Pinus merkusii* Jungh Et De Vriese	*Pinaceae*	Cone/Flower	Copper	Phenolic groups	Phenolic groups	NS	NS	FTIR, SEM	ND	20–35	ND	[53]
*Juniperus communis* L.	*Cupressaceae*	Berry/Cone	Gold	Carbonyl, Carboxyl and Hydroxyl groups	Polyphenols	24 h	RT	AFM, ATR-FTIR, EDX, UV–Vis, TEM,	Sphere and Triangle	10–50	ND	[54,55]
*Pinus densiflora*	*Pinaceae*	Cone	Silver	Hydroxyl and Carbonyl groups	Phytochemicals	15 min	RT	UV–Vis, TEM, FTIR, XRD	Oval and Triangular	40–70	ND	[56]
*Pinus merkusii* Jungh Et De Vriese	*Pinaceae*	Cone	Silver	Phenolic hydroxyl groups	Cone flower	60 min	60 °C	UV–Vis, TEM, FTIR	Sphere	8–23	ND	[57]
*Araucaria heterophylla*	*Araucariaceae*	Gum	Silver	Gum extract	Gum extract	NS	NS	AFM, EDX, FTIR, SEM, UV–Vis	ND	<25	ND	[48]
*Araucaria heterophylla*	*Araucariaceae*	Gum	Silver	Gum extract	Gum extract	NS	RT	UV–Vis, FTIR, SEM, EDX, AFM	Sphere	<30	ND	[58]
*Taxus baccata*	*Taxaceae*	Leaf/Needle	Gold	Needle extract	Needle extract	24 h	25 °C	UV–Vis, HRTEM, EDX, AFM	Sphere, Semi-sphere, Hexagonal and Triangle	<20	Several months	[59]
*Thuja orientalis*	*Cupressaceae*	Leaf/Needle	Gold	Phytochemicals	Phytochemicals	10 min	RT	EDX, FTIR, XRD, TEM, UV–Vis,	Sphere	5–94	ND	[60]
*Pinus eldarica*	*Pinaceae*	Leaf/Needle	Iron Oxide	Leaf extract	Leaf extract	30 min	RT	TEM, FTIR, XRD, EDX	Sphere	8–34	ND	[61]
*Cupressus goveniana*	*Cupressaceae*	Leaf/Needle	Silver	Carboxyl and Hydroxyl and Amine groups	Phytochemicals	60 min	80 °C	UV–Vis, FTIR, SEM	Sphere	67–200	4 months	[62]
*Cupressus sempervirens*	*Cupressaceae*	Leaf/Needle	Silver	Carbonyl and Hydroxyl groups	Phytochemicals	12 h	RT	UV–Vis, TEM, XRD, FTIR	Sphere, Hexahedral, Oval and Triangle	10–80	ND	[63]
*Cupressus torulosa* D. Don	*Cupressaceae*	Leaf/Needle	Silver	Leaf extract	Leaf extract	24 h	NS	UV–Vis, SEM, XRD, TEM	Sphere	NS	ND	[64]
*Juniperus procera*	*Cupressaceae*	Leaves	Silver	Phenolic acids, chlorogenic acid, flavonoids, caffeoylquinic acids	Phytochemicals	24 h	RT	UV–Vis SEM, FTIR	Spherical, cubical	30–90	ND	[65]
*Juniperus chinensis*	*Cupressaceae*	Leaf/Needle	Silver	Proteins	Phytochemicals	60 min	100 °C	EDX, XRD, HRTEM, UV–Vis	NS	18–25	ND	[46]
*Taxus baccata*	*Taxaceae*	Leaf/Needle	Silver	Proteins and Terpenoids	Phytochemicals	NS	10 and 30 °C	UV–Vis, TEM, AFM	Triangular and Hexagonal	75.1	Six months	[34]
*Thuja occidentalis* L.	*Cupressaceae*	Leaf/Needle	Silver	Carbonyl, Carboxyl, Aliphatic and Aromatic amine groups	Carbonyl, Carboxyl, Aliphatic and Aromatic amine groups	35–40 min	60 °C	FTIR, UV–Vis, SEM, XRD, TEM	Sphere	<30	ND	[66]
*Thuja occidentalis*	*Cupressaceae*	Leaf/Needle	Silver	Hydroxyl and Carbonyl groups	Phytochemicals	3–4 h	RT	XRD, FTIR	ND	41.48	ND	[67]
*Thuja occidentalis*	*Cupressaceae*	Leaf/Needle	Silver	Leaf extract	Leaf extract	3–4 h	RT	UV–Vis, FTIR	ND	ND	ND	[68]
*Thuja occidentalis*	*Cupressaceae*	Leaf/Needle	Silver	Leaf extract	Leaf extract	NS	NS	UV–Vis, XRD, HRTEM	ND	7–14	ND	[69]
*Thuja occidentalis*	*Cupressaceae*	Leaf/Needle	Silver	Leaf extract	Leaf extract	NS	RT	UV–Vis, XRD, TEM	ND	10–15	ND	[70]
*Torreya nucifera*	*Taxaceae*	Leaf/Needle	Silver	Proteins	Proteins	24 hrs	20 °C	FTIR, XRD, TEM, UV–Vis,	Sphere	10–125	ND	[71]
*Taxus baccata*	*Taxaceae*	Leaf/Needle	Zinc Oxide	Proteins	Proteins	NS	NS	UV–Vis, SEM, TEM, FTIR	Hexagonal	20–27.64	ND	[72]
*Pinus kesiya*	*Pinaceae*	Pollen	Gold	Pollen extract	Pollen extract	60 min	RT	UV–Vis	ND	ND	3 months	[73]
*Pinus wallichiana*	*Pinaceae*	Stem	Silver	Hydroxyl groups	Phytochemicals	30 min	55 °C	EDX, FTIR, SEM, UV–Vis, XRD	Sphere	10–30	ND	[74]
*Abies spectabilis*	*Pinaceae*	NS	Gold	Polyphenols	Polyphenols	24 h	29 °C	AFM, DLS, EDX, FTIR, UV–Vis, XRD,	Sphere	20–200	ND	[47]
*Thuja occidentalis*	*Cupressaceae*	NS	Silver	Carbonyl groups	Flavonoids, Terpenoids and Thiamines	10 min	27 °C	DLS, TEM, XRD, UV–Vis,	Sphere	122.8	ND	[75]

NS—not specified; AFM—Atomic force microscopy; ATR-FTIR—Attenuated total reflection-Fourier-transform infrared spectroscopy; DLS—Dynamic light scattering; EDX—Energy-dispersive X-ray spectroscopy; FESEM—Field emission electron microscope; HRTEM—High-resolution transmission electron microscopy; FT-IR—Fourier-transform infrared spectroscopy; RT—room temperature; SEM—Scanning electron microscopy; TEM—Transmission electron microscopy; UV–Vis—Ultraviolet-visible spectroscopy; XRD—X-ray powder diffraction. ND—Not determined.

**Table 2:** Agricultural and biomedical applications of conifer plant extract derived metal (Ag and Au) nanoparticles.

Types of Conifer	Family	Applications	Ref.
**Silver NPs**			
*Juniperus chinensis*	*Cupressaceae*	Antibacterial activity against *B. subtilis*, *E. coli*, *P. aeruginosa*, and *S. aureus*	[46]
*Juniperus procera*	*Cupressaceae*	Antimicrobial, cellular proliferation/cytotoxicity	[65]
*Pinus densiflora*	*Pinaceae*	Antibacterial activity against *Bacillus cereus*, *Brevibacterium linens*, *Propionibacterium acnes* and *Staphylococcus epidermidis*	[56]
*Pinus thunbergii*	*Pinaceae*	Antibacterial activity against *Bacillus thuringiensis*, *Bacillus megaterium*, *Burkholderia glumae*, *Pseudomonas syringae* and *Xanthomonas oryzae*	[81]
*Pinus wallichiana*	*Pinaceae*	Antibacterial activity against *Acinetobacter baumannii*; Antioxidant activity; Antipyretic activity	[74]
*Taxus baccata*	*Taxaceae*	Anti-cancerous activity against human breast (MCF-7) cell line	[72]
*Taxus yunnanensis*	*Taxaceae*	Antibacterial activity against *E. coli*, *S. aureus*, *Salmonella paratyphi* and *B. subtilis*; Anti-cancerous activity against SMMC-7721, A549, LS174T andMCF-7 cell line	[30]
*Thuja occidentalis*	*Cupressaceae*	Plant growth promoter and soil conditioner	[79]
*Thuja occidentalis*	*Cupressaceae*	Antibacterial activity against *B. subtilis*, *E. coli*, and *Pseudomonas putida*; Antifungal activity against *Aspergillus niger*, *Alternaria alternata* and *Fusarium* spp.	[66]
*Thuja occidentalis*	*Cupressaceae*	Antibacterial activity against *E. coli*, *S. aureus*; Anti-cancerous activity against HeLa, MDA-MB 231, and MCF 7 cell line	[69]
*Thuja occidentalis*	*Cupressaceae*	Antibacterial activity against *B. subtilis*, *S. aureus*, *S. typhimurium*, *L. monocytogenes* and *P. aeruginosa*;	[70]
**Gold NPs**			
*Abies spectabilis*	*Pinaceae*	Anti-cancerous activity against bladder cancer (T24) cell line	[47]
*Pinus kesiya*	*Pinaceae*	Antifungal activity against *Candida albicans*	[73]
*Taxus baccata*	*Taxaceae*	Anti-cancerous activity against MCF-7, HeLa and Caov-4 cell lines	[59]

NS—not specified.

## Abbreviations

A549	Human Lung Carcinoma Epithelial Cells
ABTS	2,2’-azino-bis(3-ethylbenzothiazoline-6-sulfonic acid)
AChE	Acetylcholinesterase
AFM	Atomic force microscopy
Ag+	Silver Ions
AgNPs	Silver Nanoparticles
ATR-FTIR	Attenuated total reflection-Fourier-transform infrared spectroscopy
AuNPs	Gold Nanoparticles
BChE	Butyrylcholinesterase
Caov-4	Human Ovarian Cancer Cell Line
CSH	*Cupressus Sempervirens* Var. Horizantalis
CSP	*Cupressus Sempervirens* Var. Pyramidalis
CuNPs	Copper Nanoparticles
DLS	Dynamic Light Scattering
DNA	Deoxyribonucleic Acid
DPPH	2,2,1-diphenyl-1-picrylhydrazyl
EDX	Energy-dispersive X-ray spectroscopy
Eos	Essential Oils
FeNPs	Iron Nanoparticles
FESEM	Field Emission Electron Microscope
FT-IR	Fourier-Transform Infrared Spectroscopy
GSH	Glutathione
GSNPs	Green Synthesized Nanoparticles
H_2_O_2_	Hydrogen peroxide
HEK293	Human Embryonic Kidney Cell Line
HeLa	Human Cells Derived From Cervical Cancer Cell Line
HRTEM	High-Resolution Transmission Electron Microscopy
HSA	Human Serum Albumin
HT1080	Human Fibrosarcoma Cell Line
IC50	Half Maximal Inhibitory Concentration
K562	Immortalised Myelogenous Leukemia Cell Line
KB	Ubiquitous KERATIN-Forming Tumour Cell Line Hela
L929	Mouse Fibroblast Cell Line
LS174T	Human Caucasian colon adenocarcinoma cell line
MDA-MB231	Epithelial, Human Breast Cancer Cell Line
MCF-7	Breast Cancer Cell Line
MIC	Minimum Inhibitory Concentration
MNPs	Metal Nanoparticles
MTT	3-(4,5-dimethylthiazol-2-yl)-2,5-diphenyltetrazolium bromide
ND	Not determined
NPs	Nanoparticles
NS	Not Specified
O2^−^	Superoxide Ion
OH•	Hydroxyl Radical
PBMCs	Peripheral Blood Mononuclear Cells
Pd/RPN	Palladium/Residue of the Pine Needle
ROS	Reactive Oxygen Species
RT	Room Temperature
SEM	Scanning Electron Microscopy
SMMC-7721	Hepatocellular Carcinoma Cell Line
TEM	Transmission Electron Microscopy
TYRO	Tyrosinase
UV–Vis	Ultraviolet-Visible Spectroscopy
WHO	World Health Organization
XRD	X-Ray Powder Diffraction
ZnO NPs	Zinc Oxide Nanoparticles
ZVI@AgNPs	Zero Valent Iron Coated Silver Nanoparticles
